# Smoothie Drinks: Possible Source of Resistant and Biofilm-Forming Microorganisms

**DOI:** 10.3390/foods11244039

**Published:** 2022-12-14

**Authors:** David Šilha, Petra Syrová, Lenka Syrová, Jana Janečková

**Affiliations:** 1 Department of Biological and Biochemical Sciences, Faculty of Chemical Technology, University of Pardubice, Studentská 573, 532-10 Pardubice, Czech Republic; 2 Department of Infectious Diagnostics, Hospital of the Pardubice Region, Jana Evangelisty Purkyně 652, 570-14 Litomysl, Czech Republic

**Keywords:** antibiotic resistance, biofilm, coliforms, enterococci, fresh bar, fungi, microbial quality, smoothie, yeast

## Abstract

Smoothie drinks are currently very popular drinks sold especially in fast food establishments. However, smoothies are a significant source of microorganisms. The aim of this study was to evaluate the microbiological quality of smoothies purchased in Eastern Bohemia. A higher prevalence of mesophilic aerobic bacteria (5.4–7.2 log CFU/mL), yeast (4.4–5.9 log CFU/mL) and coliform bacteria (3.1–6.0 log CFU/mL) was observed in vegetable smoothies, in which even the occurrence of enterococci (1.6–3.3 log CFU/mL) was observed. However, the occurrence of *S. aureus*, *Salmonella* spp. and *Listeria* spp. was not observed in any samples. Nevertheless, antimicrobial resistance was observed in 71.8% of the isolated strains. The highest level of resistance was found in isolates from smoothie drinks with predominantly vegetable contents (green smoothie drinks). Considerable resistance was observed in Gram-negative rods, especially to amoxicillin (82.2%) and amoxicillin with clavulanic acid (55.6%). Among enterococci, only one vancomycin-resistant strain was detected. The vast majority of isolated strains were able to form biofilms at a significant level, which increases the clinical importance of these microorganisms. The highest biofilm production was found in *Pseudomonas aeruginosa*, *Kocuria kristinae* and *Klebsiella pneumoniae*. Overall, significant biofilm production was also noted among isolates of *Candida* spp.

## 1. Introduction

Every day, hundreds of thousands of people around the world are endangered by unsafe food. There are a number of potential risks associated with food contamination [1]. A contaminant can be defined as a substance that has not been intentionally added to food and that occurs in that food as a result of production (including operations carried out during crop production, animal husbandry and veterinary medicine), processing, preparation, treatment, packaging, transport or environmental influences [2]. Microbial contamination poses a significant risk to human health. Microbial agents can cause serious foodborne illnesses, which, in some cases, can result in death. Symptoms of the disease can range from mild gastroenteritis to neurological, liver or kidney syndromes. More than 90% of foodborne diseases are caused by members of the *Staphylococcus*, *Salmonella*, *Clostridium*, *Campylobacter*, *Listeria*, *Bacillus* and *Vibrio* genera and some strains of *E. coli* [3].

Fresh fruit and vegetable products are often not subjected to such technological interventions that would ensure the inactivation of pathogenic microorganisms or their effective removal before consumption. However, the increasingly popular support for a healthy lifestyle has led to an increase in the consumption of fresh products [4]. Due to their high contents of natural sugars and nutrients and high water activity, fruit and vegetable drinks are prone to the greater development of some undesirable microorganisms. From these drinks, it is possible to isolate, in particular, types of microorganisms that are adapted to a highly acidic environment (yeasts, fungi and lactic acid bacteria). A potential risk is also the presence of pathogenic species, especially psychrotrophic ones (e.g., *Listeria monocytogenes*) [5]. In particular, there may be a risk of microbiological contamination at street sale points. The raw materials used for the production of fresh drinks are difficult to protect from dust and insects in these places, which can be a source of microorganisms that are harmful to human health. Similarly, water used for the production of beverages can be a significant source of microbial contamination, mainly including coliform bacteria, yeasts, fungi, streptococci and other microorganisms [6]. Foods rich in carbohydrates and lipids are also often susceptible to contamination by fungi (*Aspergillus*, *Fusarium* and *Penicillium*). Some of these fungi produce toxic mycotoxins [2,7].

Fruits and vegetables tend to be colonized by diverse microflora. Their contamination can occur during cultivation, the harvest period, post-harvest handling, processing and distribution. However, contamination can also occur through fertilization or watering, or through the transfer of microorganisms from wild animals. Microbial risks and sources of contamination can vary significantly, e.g., depending on the environment, crop type or production practices [8,9,10]. 

Microorganism resistance has been increasing considerably in recent years and is becoming a global problem. According to WHO reports in recent years, resistance to antimicrobial substances is one of the main global threats in the spectrum of infectious diseases. Worldwide research has revealed a significant increase in bacterial resistance to all groups of antibiotics. The impact of antibiotic resistance and biofilm formation is therefore not only clinical but also economic and societal [11,12]. Biofilm formation is a key virulence factor for many microorganisms. A microbial biofilm can be defined as a community of microbial cells surrounded by their own matrix of polymeric substances, which are required for cellular attachment to both biotic and abiotic surfaces. The vast majority of the volume of the biofilm is made up of exopolysaccharides, whereas only 15–20% of the volume is made up of microbial cells [13]. There are single-species and more often multi-species biofilms [14]. Bacteria in the form of a biofilm contribute, among other things, to the chronicity of persistent infections. This cell lifestyle also allows pathogens to evade the host’s immune response and thus resist antibacterial treatment. Biofilms are up to 1000 times more resistant to inhibitory effects compared to their planktonic forms [15,16].

The aim of this study was to evaluate the occurrence of microorganisms and their level of resistance to antibiotics and antifungals used in clinical practice. All strains were also evaluated in terms of their ability to form biofilm structures, which significantly increases the clinical relevance of the given strain.

## 2. Materials and Methods

### 2.1. Analysed Samples

Samples of smoothie drinks were purchased from five fresh bars during the spring months of 2022 in different cities of the Czech Republic (Pardubice, Hradec Králové). The selection of the purchased smoothie drinks was based on the content of the profile ingredients: 6 samples had predominantly vegetable components, and 4 samples had predominantly fruit components. For the purposes of the study, the samples were marked with the letter **F** for a drink with predominantly fruit components and letter **V** for a drink with predominantly vegetable components (Table 1). The second letter of the designation (A–F) indicates the establishment where the sample was obtained (e.g., the sample designation **FB** means the fruit smoothie was purchased at Fresh Bar B). The samples were transported to the laboratory in sterile containers immediately after their preparation and kept refrigerated during transport. Subsequently, the samples were immediately analysed in the laboratory.

### 2.2. Microbiological Testing

According to preliminary experiments, a dilution series from 10^−1^ to 10^−5^ in physiological saline with peptone was prepared from each sample. Selected microbiological indicators, including the total number of mesophilic aerobic bacteria, enterococci, yeasts and moulds, coliform bacteria, *Escherichia coli* and *Staphylococcus aureus* and also evidence of *Salmonella* spp. and *Listeria monocytogenes*, were evaluated.

The determination of coliform bacteria and *Escherichia coli* was performed using the selective and differentially chromogenic medium Chromocult Coliform Agar (Merck, Darmstadt, Germany; 37 °C for 24 h). For the purposes of this study, the determination of the total number of mesophilic aerobic bacteria on Plate Count Agar (Sigma-Aldrich, St. Louis, MO, USA; 30 °C for 48 h), the determination of the total number of yeasts and fungi on Dichloran Rose Bengal Chloramphenicol Agar (Merck, Darmstadt, Germany; 25 °C for 5 days), the determination of the number of enterococci on Slanetz–Bartley agar (Oxoid Ltd., Basingstoke, UK; 37 °C for 48 h) and the determination of coagulase-positive staphylococci using Baird–Parker agar medium (Sigma-Aldrich, St. Louis, MO, USA); 37 °C for 48 h) were also performed. For the detection of *Listeria monocytogenes*, primary multiplication was carried out in half broth according to Fraser (Sigma-Aldrich, St. Louis, MO, USA; 30 °C for 24 h). After primary enrichment, 0.1 mL of the culture was transferred to 10 mL of secondary enrichment Fraser medium (Sigma-Aldrich, St. Louis, MO, USA; 37 °C for 24 h) with the subsequent inoculation of both primary and secondary multiplications on ALOA and PALCAM agar (Merck, Darmstadt, Germany; 37 °C for 48 h). The detection of *Salmonella* spp. involved primary non-selective propagation in buffered peptone water (37 °C for 24 h). After incubation, the obtained culture was inoculated (secondary multiplication) into RVS broth (0.1 mL; 41.5 °C for 24 h) and MKTTn broth (1 mL; 37 °C for 24 h). Subsequently, the culture was inoculated onto selective agar media XLD and RAMBACH agar (Merck, Darmstadt, Germany; 37 °C for 24 h). All analyses were performed in duplicate and repeated twice independently. The resulting values are means with the expressed standard deviations (SDs).

### 2.3. Identification of Isolates by MALDI-TOF MS

Five macroscopically identical microbial colonies isolated from each sample/culture medium, as part of the analyses mentioned in Chapter 2.2, were sub-cultured on Mueller–Hinton agar (37 °C, 24–48 h) with subsequent identification using matrix-assisted laser desorption/ionization–time-of-flight (MALDI-TOF) mass spectrometry (MS). Each strain for identification was transferred to the target steel plate in the form of a thin film followed by overlaying 1 μL of the matrix (α-cyano-4-hydroxycinnamic acid). After drying, the plates were prepared for analysis in a MALDI Biotyper Sirius System (Bruker Daltonics GmbH, Bremen, Germany) in linear positive ion mode over the m/z range of 2000 to 20,000. Based on the TOF information, a characteristic spectrum was generated using MBT Compass Software (MBT Compass Library Revision H 2021). An achieved identification score of 2.00–3.00 represents a high-confidence identification (species level), 1.70–1.99 represents a low-confidence identification (genus level) and a score of 0–1.69 represents an unsuccessful strain identification.

### 2.4. Monitoring of Antibiotic Resistance Level

The susceptibility of isolates to amoxicillin (AMO, 10 μg), amoxicillin/clavulanic acid (AMC, 30 μg), ampicillin (AMP, 2 μg), cefepime (CPM, 30 μg), cefotaxime (CTX, 5 μg), clarithromycin (CLA, 15 μg), clindamycin (CLI, 2 μg), clotrimazole (CLO, 10 μg), colistin (COL, 10 μg), cotrimoxazole (COT, 25 μg), cefpodoxime (CPD, 10 μg), cefuroxime (CRX, 30 μg), ciprofloxacin (CIP, 5 μg), doxycycline (DOX, 30 μg), econazole (ECO, 10 μg), linezolid (LIN, 10 μg), natamycin (NAT, 50 μg), nystatine (NYS, 100 μg), ofloxacin (OFL, 5 μg), oxacillin (OXA, 30 μg), penicillin (PNC, 1 μg), ticarcillin/clavulanic acid (TIM, 85 μg) and vancomycin (VAN, 5 μg) was tested by a previously described disk diffusion method [17,18] using antimicrobial disks purchased from Oxoid Ltd. (Basingstoke, UK), Bioanalyse Ltd. (Ankara, Turkey) and ITEST plus s.r.o. (Hradec Králové, Czech Republic).

Briefly, five suspected colonies of each isolate (*n* = 85) were grown on Muller–Hinton agar, then a bacterial suspension was prepared in physiological saline, and the turbidity was adjusted according to the McFarland scale (No. 0.5) using a DEN-1 densitometer (Biosan, Riga, Latvia). Bacterial suspensions were spread onto Mueller–Hinton E agar or Mueller–Hinton 2 agar with 5% horse blood and 20 mg/L β-nicotinamide adenine dinucleotide (β-NAD) (bioMérieux, Marcy-l’Étoile, France), and yeast cultures were spread onto Mueller–Hinton agar supplemented with glucose and methylene blue (LabMediaServis, Jaroměř, Czech Republic). Antimicrobial disks were then placed on the inoculated agar medium and were incubated. Following incubation, antibiograms and minimal inhibitory concentrations (MICs) were evaluated using a BACMED 6iG2 automated reader and analyser (Aspiag, Litomyšl, Czech Republic). Isolates were classified as resistant (R), susceptible/increased exposure (I) or susceptible (S) based on breakpoint values according to CLSI and EUCAST standards [17,18].

### 2.5. Monitoring of Biofilm Formation Ability

The biofilm formation of isolated strains was monitored in flat-bottomed microtiter plates (SPL Life Sciences Co., Ltd., Pocheon-si, Republic of Korea) as previously described [19]. Briefly, 100 µL of the cell suspension (10^7^ CFU/mL) in brain heart infusion broth (BHI; Himedia, Mumbai, India) was inoculated into microtiter plates. After incubation at 30 °C for 24 h, the wells were rinsed thoroughly five times with sterile distilled water and dried. Biofilm fixation was performed with 2% sodium acetate (15 min), and biofilm-forming cells were stained with 100 µL of 1% crystal violet (Sigma-Aldrich, St. Louis, MO, USA) for 15 min. Subsequently, the unbound crystal violet was washed out carefully with sterile distilled water. Thereafter, the biofilm-associated crystal violet was solubilized with 96% ethanol. Then, 100 µL was taken from each well, and the absorbance was measured in a new plate at 595 nm (Infinite M200, Tecan, Männedorf, Switzerland). There were 8 wells in each experiment, and the experiments were independently repeated 3 times. The level of biofilm formation was categorized, according to a previously described classification system [20,21], as non-adherent (OD ≤ OD_C_) or biofilm-forming strains (OD > OD_C_), where OD_C_ (*cut-off* OD) is defined as three standard deviations above the mean OD of the negative control (blank value).. The measured and calculated OD/OD_C_ (0.111/0.120) values were the same for all measurements.

## 3. Results and Discussion

### 3.1. Microbial Quality of Smoothie Drinks

Today, it is widely recognized that fresh fruits and vegetables are a significant source of many bacteria and viruses, often with pathogenic potential [22]. This is mainly due to their considerable water activity, but also the contents of carbohydrates and other nutrients [23,24]. Foods made from fresh fruits and vegetables are popular due to their contents of natural nutrients and bioactive compounds with beneficial effects on the human organism [25]. However, a certain microbiological danger can be expected from drinks made from fresh fruits and vegetables, as there is usually a lack of technological interventions leading to the elimination of unwanted microorganisms due to the preservation of the biological activity of the drink [26,27]. The preparation of these drinks in various street establishments without strict adherence to the necessary level of hygiene and sanitation is also risky in this regard. The microbiological quality of food prepared under street conditions has already been the subject of many studies [6,28,29].

Table 2 summarizes the results of monitoring selected microbiological indicators for fruit smoothie drinks. In these smoothie drinks, the total number of mesophilic aerobic bacteria was determined to be in the range of 4.4–5.3 log CFU/mL, and the occurrence of yeast was in the range of 4.3–6.1 log CFU/mL. The occurrence of coliform bacteria was also observed in three samples (75%) of fruit smoothies. The occurrence of coliform bacteria was at a relatively low level (1.5–2.1 log CFU/mL) in our study compared to values of 2.0–4.2 log CFU/mL in an earlier study focused on the quality of smoothie drinks in Slovakia [30]. Only one sample (FC) did not contain any coliform bacteria or enterococci, which was a sample composed strictly of fruit (banana, kiwi, pear and pineapple). Enterococci, *S. aureus* and fungi were not detected in any fruit smoothie samples. Pathogenic bacteria *Salmonella* spp. and *Listeria monocytogenes* were also not detected.

The results of the microbiological evaluation of smoothie drinks with predominantly vegetable components are shown in Table 3. In the case of these samples, no *Listeria*, *Salmonella* or *S. aureus* was observed. The total number of mesophilic aerobic bacteria and the number of yeasts ranged from 5.4 to 7.2 log CFU/mL and 4.4 to 5.9 log CFU/mL, respectively. The presence of indicator microorganisms, namely, coliform bacteria (3.1–6.0 log CFU/mL) and/or enterococci (1.6–3.3 log CFU/mL), was detected in all samples. Overall, the worst microbiological quality was found in the sample labelled VA1 (cucumber, spinach, mint, apple and pineapple), in which the presence of coliform bacteria, enterococci and yeasts and fungi was detected at levels of 6.0 ± 0.05, 3.3 ± 0.09, and 8.0 ± 0.08 log CFU/mL, respectively, while the highest number of mesophilic aerobic bacteria (7.2 log CFU/mL) was also observed in this sample.

European legislation only defines a threshold limit for the occurrence of *E. coli* and *Salmonella* spp. in smoothie drinks. According to Commission Regulation (EC) No. 2073/2005 of 15 November 2005 on microbiological criteria for food, specifically according to categories 1.20 and 2.5.2, unpasteurized fruit and vegetable juices (intended for direct consumption), there must be an absence of *Salmonella* spp. in a 25 mL sample and a limit of 1000 CFU/mL in the beverage for *E. coli*. All samples included in this study thus met the prescribed hygiene limits according to Commission Regulation (EC) No. 2073/2005. However, the potential occurrence of microorganisms of concern (*Listeria monocytogenes*, *Escherichia coli*, etc.) in raw products has already been documented in detail in the past [31]. Many earlier studies also report the significant occurrence of, e.g., enterobacteria in smoothie drinks at levels of 1.9 log CFU/mL [32], 2.4 log CFU/mL [33], 3.39 log CFU/mL [34] and even 5.5 log CFU/mL [35]. The analysis of ten smoothie samples purchased in the Czech Republic revealed the presence of coliform bacteria in the range of 1.5–6.0 log CFU/mL, which basically corresponds to previously published results of studies from Argentina and Spain [32,33,35]. *Enterobacter bugandensis*, *Klebsiella variicola* and *Klebsiella pneumoniae* were among the most frequently detected representatives of coliform bacteria. The presence of the indicator bacteria *E. coli* was not detected in any of the smoothie drink samples.

In a recent study dealing with the microbiological quality of smoothie drinks in Slovakia, the presence of coliform bacteria at a level of 2.0–4.2 log CFU/mL was confirmed, which is in agreement with the conclusions of our study for all monitored samples of smoothie drinks. At the same time, however, the presence of enterococci was also observed in 90% of the samples at a level of 1.6–2.9 log CFU/mL [30]. In our experiments, enterococci were detected in only 30% of the smoothie drink samples (green smoothie drinks VA1, VA2 and VB). Most often, it was *Entrococcus mundtii*, and in one case, it was *Enterococcus casseliflavus* (see Table 3).

Microorganisms resistant to low pH values, such as yeasts and acid-tolerant microorganisms, can survive in abundance in these types of samples [36]. In all samples of smoothie drinks, the presence of yeast was detected at a density of 4.3–5.9 log CFU/mL. *Candida tropicalis*, *Candida lusitaniae* and *Candida parapsilosis* were the most frequently isolated and identified yeasts from smoothie drinks based on MALDI-TOF MS. For these non-albicans *Candida* spp., a significant prevalence and also a high level of pathogenicity have been reported [37,38,39].

Staphylococci were found in 60% of the samples, but none of the isolates were *S. aureus* or other coagulase-positive staphylococci. The most common representatives of staphylococci were *Staphylococcus epidermidis* and *Staphylococcus pasteuri*. Furthermore, the presence of *Pseudomonas aeruginosa* was also confirmed in two samples (FB and VA1).

### 3.2. Level of Antibiotic Resistance of Isolated Microorganisms

The antimicrobial resistance of microorganisms is considered one of the major threats to public health, causing serious problems in the treatment of persistent diseases [40]. In recent years, an increasing occurrence of multiresistant strains of microorganisms has been recorded in particular. Fruits and vegetables can also be a source of microorganisms carrying antibiotic resistance genes [41]. Strains isolated from smoothie drinks were evaluated in terms of their sensitivity to selected basic antimicrobial sets for testing specified groups of microorganisms according to EUCAST and CLSI, as well as according to the standard test setup in clinical practice. The results of these tests are presented in Table 4 and Table 5.

A total of 45 strains of Gram-negative rod-shaped bacteria were isolated from all samples. Resistance to amoxicillin was observed in 37 strains (82.2%). Only *Kosakonia cowanii* (one strain), *Leclercia adecarboxylata* (one strain), *Pantoea agglomerans* (five strains) and *Pantoea stewartii* (one strain) were evaluated as sensitive to amoxicillin. Amoxicillin is one of the most commonly used antibiotics in primary care. It is an aminopenicillin that was created due to increasing antimicrobial resistance. Like penicillin, it can be used against most *Streprococcus* species, and this antibiotic is also effective against *Listeria monocytogenes* and *Enterococcus* spp., but also against some strains of *Escherichia coli* [42]. Among Gram-negative rod isolates, significant resistance to amoxicillin in combination with clavulanic acid was also observed in 25 (55.6%) isolates. *Acinetobacter* was isolated from two samples, mainly vegetable smoothie drinks labelled VA1 (cucumber, spinach, mint, apple and pineapple) and VD (spinach, celeriac, lemon, apple and mango). *Acinetobacter* isolates were resistant to the greatest number of antibiotics of all Gram-negative rod isolates. *Acinetobacter baumannii* isolated from sample VA1 was even resistant to all of the tested antibiotics from different groups (penicillins, cephalosporins, fluoroquinolones, aminoglycosides and sulphonamides). Over the past 30 years, *Acinetobacter baumannii* strains have acquired resistance to a wide range of newly synthesized antimicrobial agents and have become one of the most feared pathogens worldwide [43].

Among the enterococci strains isolated from smoothie drinks, there was only one strain (25.0%) with proven resistance to co-trimoxazole and vancomycin, i.e., vancomycin-resistant *Enterococcus* (VRE). In the nine strains of staphylococci isolated, resistance was observed only to clarithromycin (55.6%) and clindamycin (11.1%). No resistance to any other antibiotics was observed. However, earlier studies specifically describe clarithromycin in combination with vancomycin as effective for the treatment of staphylococcal infections [44]. The same study also described the successful eradication of biofilm formation by combined therapy using clarithromycin and daptomycin against MRSA strains and *Staphylococcus epidermidis* strains. Similarly, reduced biofilm formation and increased drug penetration were demonstrated with the combination of clarithromycin and daptomycin [45].

Resistance to itraconazole was detected in 50.0% of isolated *Candida* spp. and *Clavispora* spp. Itraconazole is an azole antifungal that is effective against a broad spectrum of clinically relevant fungi and is used as a first-line agent for the prevention and treatment of invasive superficial infections [46]. Undoubtedly, *Candida albicans* is among the most widespread pathogenic yeasts. However, nowadays, the so-called non-albicans species of *Candida* are increasingly reported as pathogens that cause nosocomial fungal infections of the bloodstream. The most common representatives are *C. glabrata*, *C. tropicalis* and *C. parapsilosis*. *Candida tropicalis* is reported as the second most important agent that causes yeast infections [47]. In this study, *C. tropicalis* was confirmed in a total of six (60.0%) smoothie drink samples. Of this number, only two strains were sensitive to all of the tested antifungals (fluconazole, itraconazole, nystatin, clotrimazole, econazole and natamycin), and resistance to itraconazole was observed in the other strains (from samples FB, FC, FD, VA2 and VD).

Overall, it can be stated that most microbial strains were isolated from vegetable smoothie drinks, especially from those labelled VA1, VA2 and VC. Of the 15 isolates from sample VA1, 5 strains can be considered multiresistant. Similarly, 3 and 6 strains were multiresistant out of a total of 14 and 12 strains isolated from VA2 and VC samples, respectively.

### 3.3. Biofilm Formation Ability of Isolates

Microbial biofilms can be defined as a heterogeneous community of aggregated microbial cells that remain part of a matrix of extracellular polymeric substances. Biofilms can adhere to various surfaces. This virulence-enhancing property of some microorganisms can play a significant role in disease pathogenesis. Infections caused by biofilms are typically chronic in nature, as the bacteria in the biofilm structure are resistant to the immune system response and to many antimicrobials [20,48]. The ability to form a biofilm was monitored in 85 strains isolated from smoothie drink samples by using the Christensen method with microtiter plates. The results are shown in Table 4 and Table 5, and according to the usual categorization, the strains are marked as weakly adherent, moderately adherent and strongly adherent. The highest biofilm production was observed for *Pseudomonas aeruginosa* (sample FB; A = 1.8075). One more strain of *Pseudomonas aeruginosa* with significantly lower biofilm production was isolated within the study (sample VA1; A = 0.3210). *P. aeruginosa* has already been recognized in the past as one of the most life-threatening bacteria. According to a recent study, the difficulty of treating infections caused by this bacterium is caused not only by its high resistance to antibiotics but also by its significant ability to form biofilms [49].

Significant biofilm production was observed in the *Kocuria kristinae* strain (VE sample; A = 1.0259). However, there have not been many publications dealing with the formation of biofilms in this bacterium so far. Some earlier studies even describe that strains of *Kocuria* spp. are biofilm-negative bacteria [50]. However, other studies document considerable adhesion and biofilm formation in *Kocuria kristinae* strains [51].

An increased ability to form a biofilm was also noted for some strains of coliform bacteria. The highest biofilm production was observed in the strain *Klebsiella pneumoniae* (sample FB; A = 0.7581). The ability of *Klebsiella pneumoniae* to form a biofilm was also documented by earlier studies [52,53]. Among strains of *Klebsiella* spp., strongly adherent strains were recorded, but also strains with relatively low biofilm activity (A = 0.1810–0.7581). The lowest biofilm formation was observed in the *Klebsiella oxytoca* strain (sample VA1; A = 0.1336). Biofilm producers known from the literature also include *Enterobacter cloacae* and *Citrobacter freundii* [54,55,56]. *Enterobacter cloacae* strains with weak biofilm production were isolated from two samples in this study (samples VA1 and VB; A = 0.1475 and 0.1747, respectively). *Citrobacter freundii* strains were also isolated from two samples (samples VA2 and VC; A = 0.2473 and 0.2101, respectively) and were evaluated as moderately to weakly adherent strains from the point of view of biofilm formation. The occurrence of *E. coli* was not recorded in any of the samples; however, an *Escherichia hermannii* strain with increased biofilm production was isolated from the VD sample (A = 0.3802). Enterococci strains isolated from smoothie drinks were capable of biofilm formation but, in all cases, at a relatively low level.

*Staphylococcus pasteuri* (FC sample; A = 0.5714) exhibited the highest biofilm formation of all isolated staphylococci and was categorized as strongly adherent. However, other staphylococcal isolates were rated as weakly adherent. In general, staphylococci are considered important biofilm producers, which is especially true of the *Staphylococcus aureus* species, which is associated with significant health problems [57,58,59].

According to the current literature, there is no relevant information on *B. pumilus* biofilm formation [60]. However, the *Bacillus pumilus* strain (sample VE, A = 0.5528) isolated in our study was evaluated as strongly adherent and having high biofilm production. *Bacillus pumilus* was also isolated from the FD sample, but with a low ability to form a biofilm (A = 0.1426).

High levels of biofilm activity were observed among yeasts isolated from smoothie drinks. *Candida* spp. yeasts are generally known for considerable biofilm activity, which increases their virulent behaviour [61,62]. In this respect, the species *Candida albicans* is most often discussed in the literature [63]. However, some non-albicans species are capable of higher biofilm production. Out of a total of 10 yeast strains, moderate biofilm formation was recorded in 6 strains of *Candida* spp. Strong biofilm production was detected in four strains, i.e., for *Candida tropicalis* isolated from samples FB, VA2 and VD (A = 0.6680, A = 0.5998, and A = 0.7105, respectively) and *Candida lusitaniae* (A = 0.6012).

## 4. Conclusions

The trend nowadays is to eat a balanced and healthy diet. The popularity of fruit and vegetable smoothie drinks is related to this practice. However, fruits and vegetables can be a source of many microorganisms, including those dangerous to human health. Since smoothie drinks are often prepared in conditions with insufficient compliance with the necessary level of hygiene and sanitation, there is a potential risk associated with the consumption of smoothie drinks. As part of this study, the presence of mesophilic aerobic bacteria at a higher cell density, as well as of coliform bacteria, enterococci and also yeast, were detected in smoothie drinks. Even fungi were detected when analysing one of the smoothie drinks. As part of the identification of individual isolated colonies, the presence of Gram-negative rods, enterococci, staphylococci, pseudomonads, Gram-positive cocci, Gram-positive rods and bacilli was found. However, the presence of *S. aureus*, *Salmonella* spp. and *Listeria* spp. was not detected in any sample. According to Commission Regulation (EC) No. 2073/2005, all analysed samples met the criteria defined by the main standard, as the presence of *Salmonella* spp. or *Escherichia coli* was not detected in the samples. A frequently discussed problem today is the resistance of microorganisms to antimicrobial substances. The results of this study point to a significant risk of the presence of strains resistant to many antimicrobials. In some cases, they were even multiresistant strains. High resistance of Gram-negative rods was observed, especially to amoxicillin, either alone or in combination with clavulanic acid. The highest resistance was observed in the *Acinetobacter baumannii* strain, in which alarming resistance to all monitored groups of antibiotics was detected. Similarly, considerable resistance was also found for isolates of *Candida* spp. The formation of biofilms is one of the most important factors in the virulence of microorganisms, and this ability was described in basically all isolated strains of microorganisms in this study. Some strains of *Pseudomonas aeruginosa*, *Kocuria kristinae* and *Klebsiella pneumoniae* have been found to form very strong biofilms, which drastically increases the clinical significance of strains isolated from smoothie drinks.

Despite the relatively small number of samples included in this study, it seems evident that smoothie drinks can be a source of highly resistant and biofilm-forming strains of microorganisms. It is clearly necessary to pay attention to the proper observance of the level of hygiene and sanitation, even more so for the preparation of food and drinks without preservative interventions or without the addition of antimicrobial substances.

## Figures and Tables

**Table 1:** Smoothie drink samples and their compositions.

Fresh Bar A ^1^	
FA	Strawberry, mint, apple, lime
VA1	Cucumber, spinach, mint, apple, pineapple
VA2	Spinach, orange, mango, banana
Fresh Bar B ^1^	
FB	Banana, strawberries, orange
VB	Avocado, mango, spinach, apple
Fresh Bar C ^1^	
FC	Banana, kiwi, pear, pineapple
VC	Spinach, chia seeds, kiwi, mango, apple, dates, water
Fresh Bar D ^2^	
FD	Apple, orange, banana, strawberries, carrot, honey
VD	Spinach, celeriac, lemon, apple, mango
Fresh Bar E ^2^	
VE	Cucumber, spinach, mint, apple, pineapple

**F**X—fruit* smoothie drink; **V**X—vegetable* smoothie drink; *—categorization by vendor based on predominant smoothie ingredient; ^1^—Pardubice; ^2^—Hradec Králové.

**Table 2:** Occurrence of monitored microbiological indicators (log CFU/mL) in fruit smoothies.

Sample	MAB	Coliforms	Enterococci	Yeasts	Fungi	*S. aureus*	*Salmonella* spp.	*Listeria* spp.
**FA**	4.4 ± 0.00	1.6 ± 0.02	ND	4.3 ± 0.00	ND	ND	ND	ND
**FB**	5.3 ± 0.01	2.1 ± 0.05	ND	6.1 ± 0.03	ND	ND	ND	ND
**FC**	4.5 ± 0.04	ND	ND	4.8 ± 0.02	ND	ND	ND	ND
**FD**	4.5 ± 0.02	1.5 ± 0.04	ND	4.7 ± 0.05	ND	ND	ND	ND

MAB—mesophilic aerobic bacteria; ND—not detected.

**Table 3:** Occurrence of monitored microbiological indicators (log CFU/mL) in vegetable smoothies.

Sample	MAB	Coliforms	Enterococci	Yeasts	Fungi	*S. aureus*	*Salmonella* spp.	*Listeria* spp.
**VA1**	7.2 ± 0.03	6.0 ± 0.05	3.3 ± 0.09	4.4 ± 0.07	3.6 ± 0.09	ND	ND	ND
**VA2**	5.4 ± 0.8	3.4 ± 0.03	1.6 ± 0.04	5.9 ± 0.04	ND	ND	ND	ND
**VB**	6.4 ± 0.07	3.5 ± 0.05	1.8 ± 0.06	5.9 ± 0.12	ND	ND	ND	ND
**VC**	5.9 ± 0.04	3.8 ± 0.02	ND	5.6 ± 0.02	ND	ND	ND	ND
**VD**	5.4 ± 0.03	4.1 ± 0.79	ND	5.4 ± 0.09	ND	ND	ND	ND
**VE**	5.5 ± 0.07	3.1 ± 0.03	ND	5.8 ± 0.02	ND	ND	ND	ND

MAB—mesophilic aerobic bacteria; ND—not detected.

**Table 4:** Identified microorganisms isolated from fruit smoothie drink samples with their biofilm formation ability and susceptibility to antimicrobials expressed as minimum inhibitory concentration (µg/mL).

Sample	Microorganism	Score Value *	Antimicrobials						BFA
	Gram-Negative Rods		AMO	AMC	CRX	COT	DOX	CPD	
FA	*Klebsiella variicola*	2.02 **	256 (R)	4 (S)	4 (S)	0.255 (S)	1.214 (S)	0.133 (S)	MA (0.3887)
FB	*Klebsiella pneumoniae*	2.39 **	256 (R)	3.317 (S)	4 (S)	0.39 (S)	0.646 (S)	0.133 (S)	SA (0.7581)
FB	*Citrobacter farmeri*	2.41 **	256 (R)	256 (R)	256 (R)	0.048 (S)	0.646 (S)	0.25 (S)	WA (0.1529)
FB	*Enterobacter asburiae*	2.22 **	256 (R)	256 (R)	256 (R)	0.072 (S)	1.214 (S)	2 (R)	WA (0.1511)
FB	*Pantoea agglomerans*	2.31 **	0.366 (S)	0.457 (S)	0.18 (S)	0.02 (S)	0.182 (S)	0.038 (S)	WA (0.1572)
FB	*Klebsiella oxytoca*	2.32 **	256 (R)	0.94 (S)	0.94 (S)	0.11 (S)	0.344 (S)	0.038 (S)	MA (0.2569)
FC	*Pantoea septica*	2.26 **	16 (R)	0.094 (S)	8 (I)	0.072 (S)	0.344 (S)	2 (R)	WA (0.1493)
FD	*Enterobacter bugandensis*	2.27 **	256 (R)	256 (R)	256 (R)	0.11 (S)	1.214 (S)	0.94 (I)	WA (0.1503)
	Yeasts		FLU	ITR	NYS	CLO	ECO	NAT	
FA	*Candida tropicalis*	2.48 **	0.087 (S)	0.812 (I)	0.366 (S)	0.016 (S)	0.031 (S)	(S)	MA (0.3588)
FB	*Candida tropicalis*	2.51 **	0.137 (S)	2 (R)	0.415 (S)	0.021 (S)	0.218 (I)	(S)	SA (0.6680)
FC	*Candida guilliermondii*	2.62 **	0.257 (S)	2 (R)	0.117 (S)	0.125 (S)	0.125 (S)	(S)	MA (0.2779)
FD	*Candida tropicalis*	2.65 **	0.073 (S)	2 (R)	0.415 (S)	0.125 (S)	0.125 (S)	(S)	MA (0.2969)
	Staphylococci		OXA	CLI	CLA	COT	DOX	CIP	
FC	*Staphylococcus pasteuri*	2.22 **	0.049 (S)	0.087 (S)	4 (R)	0.145 (S)	0.212 (S)	0.12 (S)	SA (0.5714)
FD	*Staphylococcus epidermidis*	2.41 **	0.009 (S)	0.133 (S)	0.423 (S)	0.779 (S)	0.796 (S)	0.052 (S)	WA (0.1641)
	Pseudomonads		CTX	TIM	CPM	CIP	COL		
FB	*Pseudomonas aeruginosa*	2.34 **	256 (R)	13.548 (S)	0.412 (S)	0.087 (S)	0.5 (S)		SA (1.8075)
	Gram-positive cocci		PNC	AMC	CLA	COT	DOX		
FA	*Leuconostoc pseudomesenteroides*	2.61 **	0.115 (S)	2.732 (S)	0.001 (S)	0.801 (S)	(S)		MA (0.3107)
FB	*Leuconostoc mesenteroides*	2.60 **	0.114 (S)	0.993 (S)	0.001 (S)	8 (R)	(S)		WA (0.1505)
	Bacilli		AMC	COT	DOX				
FB	*Bacillus circulans*	2.49 **	16 (R)	16 (R)	32 (R)				MA (0.2590)
FD	*Bacillus pumilus*	2.51 **	0.001 (S)	0.033 (S)	0.055 (S)				WA (0.1426)

AMO—amoxicillin; AMC—amoxicillin/clavulanic acid; CIP—ciprofloxacin; CLA—clarithromycin; CLI—clindamycin; CLO—clotrimazole; COL—colistin; COT—cotrimoxazole; CPD—cefpodoxime; CPM—cefepime; CRX—cefuroxime; CTX—cefotaxime; DOX—doxycycline; ECO—econazole; FLU—fluconazole; ITR—itraconazole; NAT—natamycin; NYS—nystatine; OXA—oxacillin; PNC—penicillin; TIM—ticarcillin/clavulanic acid. Antimicrobial susceptibility: R—resistant strain; I—susceptible/increased exposure strain; S—susceptible strain. BFA—biofilm formation ability: WA—weakly adherent; MA—moderately adherent; SA—strongly adherent. Value in parentheses represents the actual measured absorbance value. * MALDI-TOF MS identification score values; ** species-level identification (score value > 2.0).

**Table 5:** Identified microorganisms isolated from vegetable smoothie drink samples with their biofilm formation ability and susceptibility to antimicrobials expressed as minimum inhibitory concentration (µg/mL).

Sample	Microorganism	Score Value *	Antimicrobials						BFA
	Gram-Negative Rods		AMO	AMC	CRX	COT	DOX	CPD	
VA1	*Enterobacter cloacae*	2.29 **	256 (R)	256 (R)	256 (R)	0.255 (S)	1.214 (S)	0.94 (I)	WA (0.1475)
VA1	*Siccibacter colletis*	2.36 **	256 (R)	256 (R)	256 (R)	0.167 (S)	0.344 (S)	0.47 (S)	WA (0.1120)
VA1	*Klebsiella oxytoca*	2.78 **	256 (R)	0.006 (S)	0.021 (S)	0.072 (S)	0.646 (S)	0.003 (S)	WA (0.1336)
VA1	*Klebsiella pneumoniae*	2.46 **	256 (R)	6.869 (I)	6.869 (I)	0.255 (S)	1.214 (S)	0.133 (S)	WA (0.1817)
VA1	*Providencia rettgeri*	2.41 **	256 (R)	256 (R)	0.266 (S)	0.11 (S)	256 (R)	0.002 (S)	WA (0.1124)
VA1	*Enterobacter bugandensis*	2.43 **	256 (R)	256 (R)	256 (R)	0.255 (S)	1.214 (S)	2 (R)	WA (0.1345)
VA1	*Acinetobacter baumannii*	2.49 **	256 (R)	256 (R)	256 (R)	256 (R)	256 (R)	2 (R)	WA (0.1426)
VA1	*Pantoea agglomerans*	2.53 **	0.052 (S)	0.953 (S)	0.18 (S)	0.038 (S)	0.137 (S)	0.006 (S)	WA (0.1236)
VA2	*Citrobacter freundii*	2.37 **	256 (R)	256 (R)	256 (R)	0.167 (S)	1.214 (S)	2 (R)	MA (0.2473)
VA2	*Klebsiella pneumoniae*	2.38 **	256 (R)	0.94 (S)	0.94 (S)	0.11 (S)	1.214 (S)	0.038 (S)	MA (0.3798)
VA2	*Enterobacter hormaechei*	2.48 **	256 (R)	256 (R)	256 (R)	0.072 (S)	0.646 (S)	0.94 (S)	MA (0.2775)
VA2	*Providencia rettgeri*	2.42 **	256 (R)	256 (R)	0.114 (S)	0.068 (S)	256 (R)	0.002 (S)	WA (0.1464)
VA2	*Leclercia adecarboxylata*	2.63 **	0.019 (S)	0.266 (S)	0.94 (S)	0.11 (S)	0.646 (S)	0.038 (S)	WA (0.1472)
VA2	*Pantoea stewartii*	2.50 **	0.366 (S)	0.94 (S)	0.94 (S)	0.031 (S)	0.097 (S)	0.133 (S)	WA (0.1886)
VB	*Kluyvera intermedia*	2.46 **	256 (R)	256 (R)	256 (R)	0.072 (S)	0.344 (S)	2 (R)	MA (0.3052)
VB	*Klebsiella variicola*	2.40 **	256 (R)	4 (S)	4 (S)	0.167 (S)	1.214 (S)	0.038 (S)	MA (0.3594)
VB	*Enterobacter cloacae*	2.44 **	256 (R)	256 (R)	256 (R)	0.255 (S)	2.266 (S)	2 (R)	WA (0.1747)
VB	*Citrobacter braakii*	2.40 **	256 (R)	256 (R)	256 (R)	0.072 (S)	0.646 (S)	0.47 (S)	MA (0.2649)
VB	*Enterobacter bugandensis*	2.54 **	256 (R)	256 (R)	256 (R)	0.255 (S)	1.214 (S)	0.47 (S)	WA (0.1790)
VB	*Acidovorax wautersii*	2.33 **	16 (R)	0.001 (S)	0.532 (I)	0.026 (S)	0.005 (S)	0.001 (S)	WA (0.1872)
VB	*Pantoea agglomerans*	2.44 **	0.029 (S)	0.075 (S)	0.94 (S)	0.02 (S)	0.097 (S)	0.038 (S)	WA (0.1505)
VC	*Enterobacter ludwigii*	2.28 **	256 (R)	256 (R)	256 (R)	0.167 (S)	2.266 (S)	2 (R)	WA (0.1575)
VC	*Citrobacter freundii*	2.27 **	256 (R)	256 (R)	256 (R)	0.167 (S)	1.214 (S)	2 (R)	WA (0.2101)
VC	*Citrobacter braakii*	2.40 **	256 (R)	256 (R)	256 (R)	0.255 (S)	1.214 (S)	2 (R)	WA (0.1858)
VC	*Achromobacter piechaudii*	2.37 **	256 (R)	256 (R)	256 (R)	0.01 (S)	0.002 (S)	2 (R)	WA (0.2300)
VC	*Kosakonia cowanii*	2.48 **	256 (R)	256 (R)	256 (R)	0.11 (S)	0.646 (S)	0.133 (S)	WA (0.1675)
VC	*Hafnia alvei*	2.44 **	256 (R)	256 (R)	16 (R)	0.11 (S)	1.214 (S)	2 (R)	MA (0.2663)
VC	*Pantoea agglomerans*	2.39 **	0.049 (S)	0.314 (S)	0.104 (S)	0.107 (S)	0.097 (S)	0.052 (S)	WA (0.1548)
VD	*Escherichia hermannii*	2.34 **	256 (R)	0.075 (S)	0.94 (S)	0.11 (S)	0.646 (S)	0.038 (S)	MA (0.3802)
VD	*Enterobacter asburiae*	2.40 **	256 (R)	256 (R)	256 (R)	0.255 (S)	4.257 (I)	2 (R)	WA (0.1961)
VD	*Enterobacter bugandensis*	2.32 **	256 (R)	256 (R)	256 (R)	0.167 (S)	2.266 (S)	0.94 (I)	WA (0.1880)
VD	*Klebsiella variicola*	2.47 **	256 (R)	6.869 (I)	3.317 (S)	0.255 (S)	1.214 (S)	0.133 (S)	MA (0.3417)
VD	*Acinetobacter* spp.	1.78 ***	256 (R)	256 (R)	256 (R)	0.014 (S)	256 (R)	2 (R)	WA (0.2026)
VD	*Pantoea agglomerans*	2.35 **	0.104 (S)	0.94 (S)	0.266 (S)	0.02 (S)	0.097 (S)	0.038 (S)	WA (0.1771)
VE	*Enterobacter ludwigii*	2.47 **	256 (R)	256 (R)	256 (R)	0.11 (S)	1.214 (S)	0.94 (I)	WA (0.1345)
VE	*Kosakonia cowanii*	2.39 **	0.005 (S)	0.024 (S)	0.005 (S)	0.022 (S)	0.104 (S)	0.001 (S)	WA (0.1455)
VE	*Pantoea agglomerans*	2.37 **	256 (R)	3.317 (S)	4 (S)	0.255 (S)	1.214 (S)	0.038 (S)	WA (0.1671)
	Yeasts		FLU	ITR	NYS	CLO	ECO	NAT	
VA1	*Candida tropicalis*	2.32 **	0.036 (S)	0.812 (I)	0.57 (S)	0.014 (S)	0.027 (S)	(S)	MA (0.2939)
VA2	*Candida tropicalis*	2.33 **	0.137 (S)	2 (R)	0.415 (S)	0.053 (S)	0.192 (S)	(S)	SA (0.5998)
VB	*Candida parapsilosis*	2.29 **	0.002 (S)	0.398 (I)	0.415 (S)	0.001 (S)	0.117 (S)	(S)	MA (0.2665)
VC	*Clavispora lusitaniae*	2.29 **	0.002 (S)	0.262 (I)	0.008 (S)	0.001 (S)	0.001 (I)	(S)	MA (0.3114)
VD	*Candida tropicalis*	2.37 **	0.257 (S)	2 (R)	1.464 (S)	0.125 (S)	0.125 (S)	(S)	SA (0.7105)
VE	*Candida lusitaniae*	2.34 **	0.002 (S)	0.398 (I)	0.033 (S)	0.001 (S)	0.001 (S)	(S)	SA (0.6012)
	Enterococci		AMP	DOX	COT	LIN	CIP	VAN	
VA1	*Enterococcus casseliflavus*	2.34 **	0.162 (S)	0.301 (S)	256 (R)	0.547 (S)	0.66 (S)	256 (R)	WA (0.1145)
VA1	*Enterococcus mundtii*	2.54 **	0.566 (S)	0.057 (S)	0.038 (S)	0.104 (S)	0.193 (S)	4 (S)	WA (0.1291)
VA2	*Enterococcus mundtii*	2.38 **	0.086 (S)	0.13 (S)	0.002 (S)	0.266 (S)	0.183 (S)	4 (S)	WA (0.1382)
VB	*Enterococcus mundtii*	2.48 **	0.162 (S)	0.301 (S)	0.274 (S)	0.314 (S)	0.183 (S)	4 (S)	WA (0.1504)
	Staphylococci		OXA	CLI	CLA	COT	DOX	CIP	
VA2	*Staphylococcus haemolyticus*	2.39 **	0.171 (S)	0.087 (S)	4 (R)	0.145 (S)	0.349 (S)	0.642 (S)	WA (0.1217)
VA2	*Staphylococcus pasteuri*	2.36 **	0.004 (S)	0.087 (S)	4 (R)	0.095 (S)	0.23 (S)	0.183 (S)	WA (0.1354)
VC	*Staphylococcus epidermidis*	2.29 **	16 (R)	0.017 (S)	4 (R)	8 (R)	0.012 (S)	0.02 (S)	WA (0.1783)
VC	*Staphylococcus warneri*	2.25 **	0.036 (S)	0.203 (S)	4 (R)	0.095 (S)	0.23 (S)	0.079 (S)	WA (0.1920)
VC	*Staphylococcus sciuri*	2.23 **	0.574 (S)	1 (R)	0.423 (S)	0.334 (S)	0.23 (S)	0.423 (S)	WA (0.1463)
VD	*Staphylococcus epidermidis*	2.25 **	0.003 (S)	0.133 (S)	0.277 (S)	0.221 (S)	0.812 (S)	0.052 (S)	WA (0.2346)
VE	*Staphylococcus pasteuri*	2.34 **	0.001 (S)	0.038 (S)	4 (R)	0.095 (S)	0.349 (S)	0.277 (S)	WA (0.1829)
	Pseudomonads		CTX	TIM	CPM	CIP	COL		
VA1	*Pseudomonas aeruginosa*	2.46 **	256 (R)	31.341 (I)	0.412 (S)	0.087 (S)	0.142 (S)		MA (0.3210)
	Gram-positive rods		PNC	AMC	CLA	COT	DOX	OFL	
VA1	*Microbacterium arborescens*	2.37 **	0.24 (R)	0.001 (S)	16 (R)	16 (R)	0.001 (S)	0.603 (I)	WA (0.1281)
VA1	*Microbacterium paraoxydans*	2.52 **	0.24 (R)	0.002 (S)	0.042 (S)	0.117 (S)	0.013 (S)	2 (R)	WA (0.1416)
VA1	*Lactobacillus* spp.	1.83 ***	4 (R)	2.603 (S)	0.94 (I)	0.153 (S)	(S)	256 (R)	WA (0.1547)
VA2	*Curtobacterium flaccumafaciens*	2.19 **	0.25 (S)	1.495 (S)	0.001 (S)	0.122 (S)	(S)	256 (R)	WA (0.1418)
VA2	*Microbacterium testaceum*	2.28 **	0.088 (S)	0.001 (S)	0.014 (S)	0.073 (S)	0.066 (S)	4 (R)	WA (0.1433)
VA2	*Carnobacterium divergens*	2.30 **	4 (R)	1.385 (S)	0.002 (S)	0.186 (S)	(S)	(S)	WA (0.2115)
VA2	*Carnobacterium maltaromaticum*	2.31 **	2 (I)	3.204 (S)	0.03 (S)	0.607 (S)	(S)	(S)	WA (0.2027)
VE	*Kocuria kristinae*	2.39 **	0.5 (R)	0.019 (S)	0.423 (S)	0.722 (S)	0.532 (S)	1 (S)	SA (1.0259)
	Bacilli		AMC	COT	DOX				
VC	*Bacillus thuringiensis*	2.19 **	16 (R)	0.94 (S)	0.055 (S)				WA (0.1541)
VE	*Bacillus altitudinis*	2.21 **	0.001 (S)	0.021 (S)	0.055 (S)				WA (0.1501)
VE	*Bacillus pumilus*	2.15 **	0.001 (S)	0.05 (S)	0.055 (S)				SA (0.5528)

AMO—amoxicillin; AMC—amoxicillin/clavulanic acid; AMP—ampicillin; CIP—ciprofloxacin; CLA—clarithromycin; CLI—clindamycin; CLO—clotrimazole; COL—colistin; COT—cotrimoxazole; CPD—cefpodoxime; CPM—cefepime; CRX—cefuroxime; CTX—cefotaxime; DOX—doxycycline; ECO—econazole; FLU—fluconazole; ITR—itraconazole; LIN—linezolid; NAT—natamycin; NYS—nystatine; OFL—ofloxacin; OXA—oxacillin; PNC—penicillin; TIM—ticarcillin/clavulanic acid; VAN—vancomycin. Antimicrobial susceptibility: R—resistant strain; I—susceptible/increased exposure strain; S—susceptible strain. BFA—Biofilm Formation Ability: WA—weakly adherent; MA—moderately adherent; SA—strongly adherent. Value in parentheses represents the actual measured absorbance value. * MALDI-TOF MS identification score values; ** species-level identification (score value > 2.0); *** genus-level identification (score value 1.70–1.99).

## Data Availability

Not applicable.
